# Overweight and obesity impair left ventricular systolic function as measured by left ventricular ejection fraction and global longitudinal strain

**DOI:** 10.1186/s12933-018-0756-2

**Published:** 2018-08-14

**Authors:** Peter Blomstrand, Peter Sjöblom, Mats Nilsson, Magnus Wijkman, Martin Engvall, Toste Länne, Fredrik H. Nyström, Carl Johan Östgren, Jan Engvall

**Affiliations:** 1 Department of Clinical Physiology, County Hospital Ryhov, Jönköping, Sweden; 2 Department of Natural Science and Biomedicine, School of Health and Welfare, Jönköping University, Jönköping, Sweden; 3 Department of Medical and Health Sciences, Linköping University, Linköping, Sweden; 4 Primary Health Care and Department of Medical and Health Sciences, Linköping University, Finspång, Sweden; 5 Futurum, Academy for Health and Care, Jönköping, Sweden; 6 Department of Internal Medicine and Department of Medical and Health Sciences, Linköping University, Norrköping, Sweden; 7 Department of Clinical Physiology, Linköping University, Linköping, Sweden; 8 Center for Medical Image Science and Visualization (CMIV), Linköping University, Linköping, Sweden

**Keywords:** Overweight, Obesity, Diabetes mellitus, Echocardiography

## Abstract

**Aims:**

Obesity is associated with type 2 diabetes mellitus, left ventricular diastolic dysfunction and heart failure but it is unclear to which extent it is related to left ventricular systolic dysfunction. The aim of the study was to explore the effects of overweight and obesity on left ventricular systolic function in patients with type 2 diabetes mellitus and a control group of non-diabetic persons.

**Methods:**

We prospectively investigated 384 patients with type 2 diabetes mellitus, and 184 controls who participated in the CARDIPP and CAREFUL studies. The participants were grouped according to body mass index (normal weight < 25 kg/m^2^, overweight 25–29 kg/m^2^, and obesity ≥ 30 kg/m^2^). Echocardiography was performed at the beginning of the study and after 4-years in the patient group.

**Results:**

Univariable and multivariable regression analysis revealed that variations in left ventricular ejection fraction, global longitudinal strain, left ventricular mass and diastolic function expressed as E/é (the ratio between early diastolic mitral flow and annular motion velocities) all are related to body mass index. The mean and standard deviation of left ventricular ejection fraction and global longitudinal strain values were 57% (8%) vs. − 18.6% (2.3%) for normal weight patients, 53% (8%) vs. − 17.5% (2.3%) for overweight, and 49% (9%) vs. − 16.2% (3.0%) for obese (p < 0.05 vs. p < 0.05). Corresponding results in the control group were 58% (6%) vs. − 22.3% (3.0%), 55% (7%) vs. − 20.8% (3.1%) and 54% (8%) − 19.6% (4.0%) (p < 0.05 vs. p < 0.05). Patients who gained weight from baseline to follow-up changed left ventricular ejection fraction (median and interquartile range) by − 1.0 (9.0) % (n = 187) and patients who lost weight changed left ventricular ejection fraction by 1.0 (10.0) % (n = 179) (p < 0.05).

**Conclusion:**

Overweight and obesity impair left ventricular ejection fraction and global longitudinal strain in both patients with type 2 diabetes mellitus and non-diabetic persons.

*Trial registration* ClinicalTrials.gov identifier NCT 01049737

## Background

The prevalence of overweight and obesity is increasing. In 2016, 39% of adults worldwide suffered from overweight and 13% from obesity [1]. Overweight is defined by WHO as a body mass index (BMI) value ≥ 25 kg/m^2^ and obesity as a BMI value ≥ 30 kg/m^2^. Obesity is associated with conditions that increase cardiovascular morbidity and mortality [e.g., lipid metabolic disturbances, type 2 diabetes mellitus (T2DM), hypertension, and coronary artery disease] [2]. Obesity is an independent predictor of left ventricular (LV) hypertrophy, diastolic dysfunction, and heart failure [3–5] but the association with systolic function is more complex. Obesity, as measured by BMI, is associated with subclinical LV systolic dysfunction measured using echocardiographic myocardial tissue signal (deformation imaging) [6–8], but also improved left ventricular ejection fraction (LVEF) [9, 10]. Abdominal obesity, as measured by waist circumference, has shown to be associated with impaired peak global longitudinal strain (GLS) [6, 10]. The extent to which obesity is associated with LV systolic dysfunction independent of the concomitant increase in other risk factors is unclear [11].

Diabetes mellitus type 2 is also associated with hypertension and coronary artery disease and is an independent risk factor for myocardial infarction [12]. LV systolic and diastolic dysfunction and hypertrophy are common in patients with T2DM [13, 14]. Multifactorial pathophysiological mechanisms are likely mediators to diabetic cardiomyopathy with systolic and diastolic LV dysfunction heralding the development of congestive heart failure, independent of coronary artery disease and hypertension. The extent to which obesity contributes to impairment of LV systolic function in patients with T2DM is unclear but it is our hypothesis that it is associated with impaired systolic function as measured by use of both LVEF and deformation imaging.

## Methods

### Study population

The aim of this study was to assess the relationship between overweight and obesity to the LV remodelling of systolic function in patients with T2DM and a control group of non-diabetic persons. We enrolled 512 consecutive patients who participated in the Cardiovascular Risk factors in Patients with Diabetes—a Prospective study in Primary care (CARDIPP) study [15]. Participants were recruited from 13 primary healthcare centres between 2005 and 2009. Except for patients with severe physical or mental disease and those with a short life expectancy (i.e., < 1 year), all patients with T2DM, 55–65 years of age who wanted to participate were included. As non-diabetic controls, we used 185 age-matched, randomly selected individuals, from a population register, the CAREFUL (Cardiovascular Reference Population) study [16]. The studies are registered with Clinicaltrials.gov, number NCT 01049737.

The investigation included collection of medical history and ongoing medical therapy data from patient records. Each person underwent an echocardiographic examination at time of enrolment into the study. Height and weight were measured. Blood pressure was measured in supine position at rest using a fully automated oscillometric device (Dinamap PRO 200 Monitor, Critikon, Tampa, FL, USA). All patients in the CARDIPP study were invited to join an echocardiographic follow-up study 4 years after the initial examination but 112 declined to participate and were therefore excluded. The echocardiographic examinations were performed at the Department of Clinical Physiology, Linköping University Hospital, Linköping, Sweden.

### Laboratory analyses

Urine and blood specimens were taken in the morning following a 10-h overnight fast. Creatinine, total cholesterol, high density lipoprotein (HDL), low density lipoprotein (LDL) and triglyceride concentrations were measured. HbA1c was analysed using a Swedish Mono-S HPLC. Since the control group specifically excluded participants with T2DM, HbA1c was not measured in CAREFUL. Renal glomerular function was assessed by eGFR. Microalbuminuria was defined according to the American Diabetes Association definition, as uACR ≥ 3.0 mg/mmol.

### Echocardiography

Echocardiography was performed using a Vivid 7 Ultrasound System (GE Vingmed Ultrasound; General Electric Milwaukee, WI, USA) for assessment of LV morphology, systolic and diastolic function. Left atrial (LA) end-systolic size, left ventricular end-diastolic diameter (LVEDD), end-diastolic thickness of the septum and posterior wall were all measured in the parasternal long axis view using M-mode tracing. The linear method and the parasternal long axis approach were used to calculate LV mass. Apical two- and four-chamber views and the apical long axis view were acquired at a frame rate of > 40 frames/s, and were analysed off-line by one experienced independent operator using 2D speckle tracking (EPPC, EchoPAC PC version 112, GE Ultrasound, Horten, Norway). Peak global longitudinal strain was calculated as the mean value from 18 segments of the left ventricle. LV end-diastolic volume (LVEDV) and LVEF were measured using the modified Simpson’s method included in the EPPC software as a manually adjusted semi-automated quantification tool (Auto-EF) [17]. Body surface area (BSA), calculated by the DuBois formula, was used to index the measurements of LA, LVEDV and LV mass. Early diastolic transmitral inflow velocity (E) was obtained at the level of the tip of the mitral leaflets using pulsed wave Doppler ultrasound. Color tissue Doppler loop images were obtained in the two- and four-chamber apical views. Mitral annular plane systolic excursion (MAPSE) was measured using the tissue tracking algorithm, which uses color tissue Doppler measurement of systolic tissue velocity, integrated over time [18]. MAPSE was calculated by averaging the total amplitude values measured from three consecutive heartbeats at the septal, lateral, inferior, and anterior aspects of the two- and four-chamber views. Peak early diastolic myocardial velocity (é) was measured from color tissue Doppler recordings at the base of the septum and lateral wall and was presented as the mean of three consecutive heartbeats [19]. The E/é ratio was calculated and was considered to reflect the filling pressures to the left ventricle [20].

### Statistical analysis

The clinical aim of the study was to assess the relationship between overweight and obesity, as measured by use of BMI, and LV systolic function in T2DM patients and non-T2DM controls. For statistical analysis, SPSSV.22.0 software (SPSS, Chicago, IL, USA) and Statistical Analysis Software, SAS/STAT (Cary, USA) were used. The results for categorical variables were presented as numbers and percentages. Continuous variables were presented as median and interquartile ranges and in analysis of variance (unbalanced ANOVA) as mean and standard deviation (SD).

Univariable and multivariable linear regression analysis were used to investigate the relationships between baseline characteristics and LV function. In the multivariable linear regression models, those explanatory variables that had estimates of the regression parameters (e.g. β_1_) that were statistically significantly different from zero together with age and gender were included. Pearson’s correlation analysis was used to examine the associations between BMI and left ventricular function and mass. To analyse the relationship between T2DM and left ventricular function, patients and controls were merged in a final regression analysis. Check for multicollinearity as well as homogeneity of variance was performed.

To assess the risk for LV systolic dysfunction in patients with overweight and obesity, odds ratios and 95 percent confidence intervals were calculated for LVEF categorized as normal or impaired (normal defined as ≥ 52% in male and ≥ 54% in female participants, according to recommendations [21] and BMI (normal, overweight and obesity). Wilcoxon signed rank test was used to analyse differences between matched samples within a group and Mann–Whitney U-test between groups. To compare participants with BMI values < 25 kg/m^2^, 25–29 kg/m^2^, and ≥ 30 kg/m^2^, variance analysis using unbalanced ANOVA was used for continuous variables. Pairwise comparisons and χ^2^ tests were used for analysis of discrete variables. Inter-observer variability was determined by use of a two-way mixed, absolute agreement, intra-class correlation (ICC) analysis, and limits of agreement and coefficient of variation. A *p* value < 0.05 was considered to indicate a statistically significant result.

## Results

Results from 16 patients and one control were excluded because of bad image quality or missing data. The study population consisted of the remaining 384 patients and 184 controls. Baseline characteristics of the enrolled participants are presented in Table 1.

**Table 1:** Baseline characteristics in patients and controls

	Patients n = 384	Controls n = 184	p
Demographic data			
Age, years	61 (5)	64 (9)	< 0.001
Female, n (%)	113 (29)	91 (49)	< 0.001
Smoker or former smoker, n (%)	257 (67)	91 (49)	< 0.001
Height, cm	174 (12)	172 (14)	ns
Weight, kg	90 (19)	76 (21)	< 0.001
BMI, kg/m^2^	30 (6)	26 (5)	< 0.001
Medical history			
Diabetes duration, years	6 (7)	0	na
Angina pectoris, n (%)	30 (8)	3 (2)	< 0.01
Myocardial infarction, n (%)	24 (6)	5 (3)	ns
Coronary revascularization, n (%)	25 (6)	4 (2)	< 0.05
Heart failure, n (%)	8 (2)	0	na
Hypertension, n (%)	253 (66)	43 (23)	< 0.001
Medication			
Loop diuretics, n (%)	26 (7)	0	na
Statins, n (%)	230 (60)	19 (10)	< 0.001
ACEI and/or ARB, n (%)	187 (49)	21 (11)	< 0.001
Calcium channel blocker, n (%)	68 (18)	9 (6)	< 0.001
β-Blocker, n (%)	138 (36)	25 (14)	< 0.001
Laboratory analyses			
HbA1c, %	5.8 (1.3)		na
Triglycerides, mmol/L	1.5 (1.0)	1.1 (0.8)	< 0.001
Triglycerides, mg/dL	133 (88)	97 (71)	< 0.001
LDL-C, mmol/L	2.5 (0.9)	3.5 (1.0)	< 0.001
LDL-C, mg/dL	96 (35)	135 (39)	< 0.001
Microalbuminuria, n (%)	60 (16)	7 (4)	< 0.001
Creatinine, µmol/L	88 (23)	77 (20)	< 0.001
Creatinine, mg/dL	1.00 (0.26)	0.87 (0.23)	< 0.001
GFR, mL/min/1.73 m^2^	70 (20)	76 (18)	< 0.001
Blood pressure (mmHg)			
Systolic blood pressure	137 (20)	128 (30)	< 0.001
Diastolic blood pressure	78 (12)	74 (15)	< 0.001

Values are presented as numbers of participants and percent for categorical variables, and median and (interquartile range) for continuous variables

*ns* not significant, *na* not applicable, *BMI* body mass index, *ACEI* angiotensin converting enzyme inhibitor, *ARB* angiotensin II receptor blocker, *HbA1c* glycosylated haemoglobin, *LDL-C* low-density lipoprotein cholesterol

The patient group had a larger proportion of men and weighed in average 14 kg more than the control group. Due to the voluntary nature of participation in the control group, they were in average 3 years older than the CARDIPP patients (Table 1). The proportion of obese participants was 45% in the patient group and 15% in the control group. The latter is very close to the level reported in the general Swedish population [22]. The T2DM patients were in general well controlled with more than eight out of ten having HbA1c values below 7%. Echocardiographic results at baseline and follow-up are presented in Table 2. The patients had higher left ventricle mass, lower LVEF and higher E/é than the controls. One hundred and nighty-one patients (50%) and 127 controls (69%) had normal LVEF, 34 (9%) patients and three controls (2%) had moderate or severe reduction of LVEF (≤ 41% is considered to be the cut-off for moderately reduced systolic LV function).

**Table 2:** Echocardiographic results in patients at baseline and at the 4-year follow-up examination, and in controls

	Patients, n	Patients baseline	Patients follow-up	p-value Patients baseline vs. follow-up	Controls, n	Controls baseline	p-value Patients baseline vs. controls
LA, mm/m^2^	380	20.4 (3.1)	20.6 (3.1)	ns	183	20.8 (4.0)	ns
LVEDV, mL/m^2^	371	46.1 (13.1)	46.5 (12.0)	< 0.05	180	48.8 (11.3)	< 0.01
LV mass, g/m^2^	324	101.8 (30.3)	101.7 (26.9)	ns	174	94.5 (29.9)	< 0.001
LVEF, %	370	52.0 (10.5)	53.0 (11.0)	ns	180	57.0 (9.0)	< 0.001
MAPSE, mm	370	12.0 (2.0)	12.0 (2.0)	ns	184	12.3 (2.3)	ns
E/é	378	11.9 (4.5)	13.3 (4.9)	< 0.001	183	10.3 (4.1)	< 0.001

Data are presented as median and (interquartile range)

*LA* LVEDV and LV mass are indexed by body surface area, *ns* not significant, *LA* left atrium, *LVEDV* left ventricular end-diastolic volume, *LV* left ventricle, *LVEF* left ventricular ejection fraction, *MAPSE* mitral annular plane systolic excursion, *E* transmitral E-wave velocity, *eʹ* early diastolic mitral annulus velocity

**Fig. 1:**
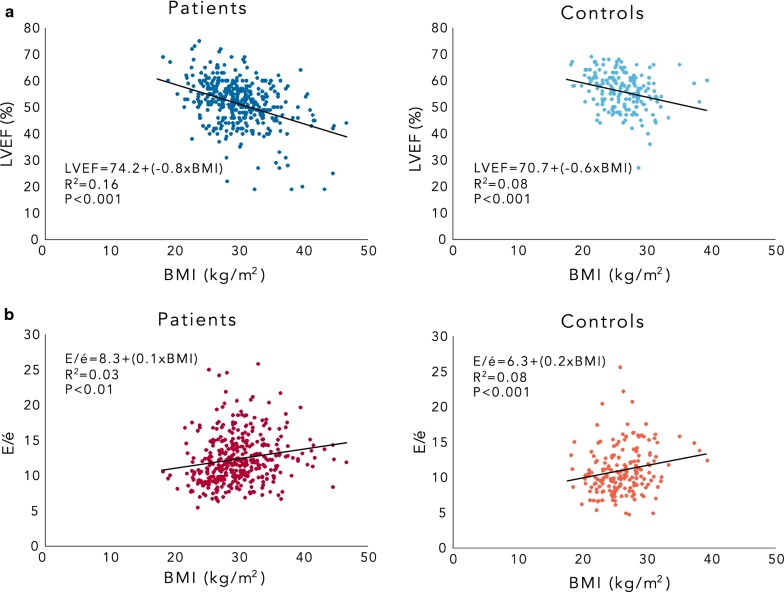
Scatterplot between BMI and LVEF versus BMI and E/é in T2DM patients and controls. Results of linear regression between left ventricular ejection fraction (LVEF) and body mass index (BMI) in patients and controls (**a**). In **b**, LV diastolic function expressed as the ratio between the peak early mitral flow velocity (E) and the mean of peak early diastolic myocardial velocity (é) measured at the base of the septum and the lateral wall is plotted vs BMI

Univariable linear regression analysis showed that BMI was associated with LV mass, LVEF, GLS and E/é in patients and LVEF, GLS and E/è in controls (Tables 3, 4) (Fig. 1). Multivariable linear regression analysis resulted in the inclusion of BMI in addition to LV mass, LVEF and GLS in the final estimated models for T2DM patients and LVEF, GLS in controls. T2DM was associated with LVEF and E/é in univariable linear regression analyses (β_1_ = − 4.5, p < 0.001, R^2^ = 0.06) vs. (β_1_ = 1.2, p < 0.001, R^2^ = 0.03) but not in the final estimated models using multivariable linear regression analyses when the two groups were merged. The odds ratio between LVEF (normal vs. abnormal) explanatory variables showed that the only variable that had any impact on LVEF was BMI. The odds ratio for an abnormal LVEF in patients with overweight was 2.2 (95% CI 1.6–3.0) and obesity 4.8 (95% CI 2.6–9.2). Correspondingly, the odds ratio for an abnormal LVEF in controls with overweight was 2.1 (95% CI 1.0–4.4) and obesity 2.1 (95% CI 0.8–5.7).

**Table 3:** Relationships between echocardiographic variables and baseline characteristics in patients, results from univariable and multivariable regression analysis

Explanatory variable	LV mass		LVEF		GLS		E/é	
	Univariable	Multivariable model R^2^ = 0.23	Univariable	Multivariable model R^2^ = 0.19	Univariable	Multivariable model R^2^ = 0.24	Univariable	Multivariable model R^2^ = 0.18
	β_1_, R^2^	**B**	β_1_, R^2^	**B**	β_1_, R^2^	**B**	β_1_, R^2^	**B**
Demographic data								
Age, years	0.4, *0.00*		0.08, *0.00*		− 0.02, *0.00*		0.14*, *0.02*	
Gender = female	− 12.8***, *0.06*	− 8.1**	− 0.04, *0.00*		− 0.91**, *0.03*	− 0.8*	1.08**, *0.02*	1.4***
Smoker/former smoker	4.2, *0.01*		− 0.8, *0.00*		− 0.27, *0.00*		− 0.30, *0.00*	
BMI, kg/m^2^	1.1***, *0.05*	1.1***	− 0.7***, *0.16*	− 0.7***	0.17***, *0.10*	0.2***	0.13**, *0.03*	
Medical history								
Diabetes duration, years	− 0.1, *0.00*		0.09, *0.00*		− 0.01, *0.00*		0.05, *0.00*	
Angina pectoris	17.9***,*0.04*		− 4.6**, *0.02*		2.7***, *0.00*	1.6***	1.22, *0.01*	
Myocardial infarction	13.2*, *0.02*		− 3.3, *0.01*		1.7**, *0.02*		0.74, *0.00*	
Coronary revascularization	16.2**, *0.02*		− 3.0, *0.01*		1.67**, *0.03*		− 0.37, *0.00*	
Heart failure	20.8*, *0.01*		− 12.2***, *0.05*	− 9.6***	4.3***, *0.05*	2.5**	4.05**, *0.03*	
Hypertension	3.8, *0.01*		1.6, *0.01*		− 0.13, *0.00*		0.60, *0.01*	
Medication								
Loop diuretics	6.8, *0.00*		− 5.8***, *0.03*		1.9**, *0.03*		2.7***, *0.04*	2.1**
Statins	− 3.3, *0.00*		− 0.59, *0.00*		0.26, *0.00*		0.28, *0.00*	
ACEI and/or ARB	4.2, *0.01*		0.38, *0.00*		0.00, *0.00*		0.60, *0.01*	
Calcium channel blocker	12.9***, *0.04*	6.6*	− 0.24, *0.00*		0.27, *0.00*		0.95*, *0.01*	
β-Blocker	12.8***, *0.06*	9.4***	0.33, *0.00*		− 0.01, *0.00*		1.02**, *0.02*	0.8
Laboratory analyses								
HbA1c, %	0.9, *0.00*		− 1.2**, *0.02*		0.55***, *0.04*	0.4**	0.33, *0.01*	
Triglycerides, mmol/L	1.2, *0.00*		− 1.4*, *0.01*		0.43*, *0.01*		0.38*, *0.01*	0.4*
LDL-C, mmol/L	− 1.2, *0.00*		− 0.32, *0.00*		− 0.05, *0.00*		− 0.15, *0.00*	
Microalbuminuria	14.8***, *0.05*	8.4**	− 1.8, *0.01*		0.49, *0.00*		1.55**, *0.03*	1.1*
Creatinine, µmol/L	0.3***, *0.04*	0.2*	0.02, *0.00*		0.01, *0.00*		0.00, *0.00*	
GFR, mL/min/1.73 m^2^	− 0.1, *0.00*		− 0.03, *0.00*		0.00, *0.00*		− 0.01, *0.00*	
Blood pressure (mmHg)								
Systolic blood pressure	0.3***, *0.03*	0.2*	− 0.02, *0.00*		0.02**, *0.02*		0.06***, *0.07*	0.06***
Diastolic blood pressure	0.2, *0.01*		− 0.05, *0.00*		0.06***, *0.03*	0.04**	0.04*, *0.01*	

Results from univariable regression Y = β_0_ + β_1_*X + ε. Results are presented as estimated regression parameters β_1_ and p-values (* p < 0.05, ** p < 0.01 and *** p < 0.001) for parameters and R^2^. Hypothesis tested H_0_: β_0_ and β_1_ = 0 against H_1_: β_0_ and β_1_ ≠ 0. Note that the β_0_ term is not presented in the table. Final results of multivariable linear regression analysis Y = **Β** X + ε of LV mass, LVEF and E/é. **B** is the estimated vector of regression parameters in the multivariable model. ε represents the error-term in the model. Results are presented as estimated regression parameters, p-values for the parameters, and R^2^, the explained proportion of variation in the outcome variable by the explanatory variables. LV mass are indexed by body surface area. See abbreviations as in Tables 1 and 2

**Table 4:** Relationships between echocardiographic variables and baseline characteristics in controls, results from univariable and multivariable regression analysis

Explanatory variable	LV mass		LVEF		GLS		E/é	
	Univariable	Multivariable model R^2^ = 0.19	Univariable	Multivariable model R^2^ = 0.09	Univariable	Multivariable model R^2^ = 0.16	Univariable	Multivariable model R^2^ = 0.22
	β_1_, R^2^	**B**	β_1_, R^2^	**B**	β_1_, R^2^	**B**	β_1_, R^2^	**B**
Demographic data								
Age, years	0.4, *0.01*		− 0.03, *0.00*		0.02, *0.00*		0.21***, *0.12*	0.2***
Gender = female	− 15.0***, *0.11*	− 10.9**	1.9, *0.02*		− 1.5**, *0.05*	− 1.0	0.5, *0.00*	1.5**
Smoker/former smoker	2.1, *0.00*		− 0.03, *0.00*		0.38, *0.01*		0.20, *0.00*	
BMI, kg/m^2^	0.8, *0.21*		− 0.56***, *0.08*	− 0.6***	0.27***, *0.01*	0.2***	0.26***, *0.08*	
Medical history								
Angina pectoris	2.9, *0.00*		− 1.0, *0.00*		− 0.85, *0.00*		3.2, *0.02*	
Myocardial infarction	11.4, *0.01*		− 2.0, *0.00*		1.2, *0.00*		− 0.46, *0.00*	
Coronary revascularization	10.1, *0.00*		0.8, *0.00*		− 0.49, *0.00*		− 3.3*, *0.02*	
Hypertension	10.4*, *0.04*		− 1.9, *0.01*		0.86, *0.01*		0.50, *0.00*	
Medication								
Statins	12.6*, *0.03*	12.9*	− 3.3, *0.02*		1.1, *0.01*		− 0.02, 0.00	
ACEI and/or ARB	8.3, *0.02*		− 2.2, *0.01*		1.2, *0.01*		− 0.44, *0.00*	
Calcium channel blocker	15.6*, *0.03*		0.5, *0.00*		− 0.81, *0.00*		0.53, *0.00*	
β-Blocker	5.6, *0.01*		− 1.0, *0.00*		0.55, *0.00*		0.13, *0.00*	
Laboratory analyses								
Triglycerides, mmol/L	0.9, *0.00*		0.06, *0.00*		0.01*, *0.00*		− 13, *0.00*	
LDL-C, mmol/L	− 1.9, *0.00*		0.34, *0.00*		0.36, *0.01*		0.40, *0.01*	
Microalbuminuria	10.5, *0.01*		− 14.8, *0.00*		11.3, *0.00*		3.5*, *0.04*	
Creatinine, µmol/L	0.3*, *0.03*		− 0.08, *0.00*		0.09, *0.02*		0.05, *0.01*	
GFR, mL/min/1.73 m^2^	0.1, *0.00*		0.04, *0.01*		0.00, *0.00*		− 0.03, *0.02*	
Blood pressure (mmHg)								
Systolic blood pressure	0.4***, *0.09*	0.3**	− 0.03, *0.01*		0.02, *0.01*		0.06***, *0.09*	
Diastolic blood pressure	0.6**, *0.08*		− 0.1, *0.02*		0.08***, *0.07*	0.2**	0.10***, *0.08*	0.1***

Results from univariable regression Y = β_0_ + β_1_ *X + ε. Results are presented as estimated regression parameters β_1_ and p-values (* p < 0.05, ** p < 0.01 and *** p < 0.001) for parameters and R^2^. Hypothesis tested H_0_: β_0_ and β_1_ = 0 against H_1_: β_0_ and β_1_ ≠ 0. Note that the β_0_ term is not presented in the table. Final results of multivariable linear regression analysis Y = **Β** X + ε of LV mass, LVEF and E/é. **B** is the estimated vector of regression parameters in the multivariable model. ε represents the error-term in the model. Results are presented as estimated regression parameters, p-values for the parameters, and R^2^, the explained proportion of variation in the outcome variable by the explanatory variables. LV mass are indexed by body surface area. See abbreviations as in Table 1 and 2

To investigate the association between weight expressed as BMI and remodelling in terms of LV size and function, the participants were divided into three groups according to BMI (Table 5). There were minor differences in demography, medical history and laboratory results between the BMI-groups. There were more female patients in the obese group compared to the overweight group. In the control group there were more females in the normal weight group compared to the overweight group. The differences between the weight groups were striking in both patients and controls. Patients and controls with elevated BMI had impaired LV systolic function, as measured by both LVEF and GLS, compared with normal weight participants (Table 5).

**Table 5:** Baseline characteristics, clinical, laboratory, and echocardiographic results in persons grouped by body mass index (BMI)

	I	II	III	Significance between groups, p < 0.05
	Normal weight	Overweight	Obesity	
	BMI < 25.0 kg/m^2^	BMI 25.0–29.9 kg/m^2^	BMI ≥ 30.0 kg/m^2^	
	Patients, n = 48	Patients, n = 163	Patients, n = 173	
	Controls, n = 73	Controls, n = 84	Controls, n = 27	
Demographic data				
Age, years				
Patients	60.5 (2.8)	60.8 (3.1)	60.7 (3.1)	ns
Controls	62.9 (5.8)	63.8 (5.7)	64.5 (5.8)	ns
Female, n (%)				
Patients	15 (31.2)	39 (23.9)	59 (34.1)	II–III
Controls	44 (60.3)	36 (42.8)	11 (40.7)	I–II
Medical history				
Diabetes duration, years				
Patients	6.4 (5.8)	6.4 (5.4)	6.8 (5.2)	ns
Controls	0	0	0	ns
Angina, n (%)				
Patients	4 (8.3)	10 (6.1)	25 (14.4)	II–III
Controls	0	3 (3.6)	0	ns
Myocardial infarction, n (%)				
Patients	3 (6.2)	9 (5.5)	12 (6.9)	ns
Controls	1 (1.4)	2 (2.4)	2 (7.4)	ns
Coronary revascularization, n (%)				
Patients	2 (4.2)	10 (6.1)	13 (7.5)	ns
Controls	1 (1.4)	1 (1.2)	2 (7.4)	ns
Heart failure, n (%)				
Patients	1 (2.1)	1 (0.6)	6 (3.5)	ns
Controls	0	0	0	ns
Hypertension, n (%)				
Patients	31 (64.6)	101 (62.0)	112 (64.7)	ns
Controls	12 (16.4)	21 (25.0)	10 (37.0)	I–III
Laboratory analyses				
HbA1c, %				
Patients	5.5 (0.9)	6.0 (1.0)	6.1 (1.0)	I–II, I–III
Triglycerides, mmol/L				
Patients	1.2 (0.6)	1.7 (0.9)	1.9 (0.9)	I–II, I–III
Controls	1.3 (1.6)	1.4 (0.6)	1.6 (0.9)	ns
Triglycerides, mg/dL				
Patients	106.2 (53.1)	150.4 (79.6)	168.1 (79.6)	I–II, I–III
Controls	115.0 (141.6)	123.9 (53.1)	141.6 (79.7)	ns
LDL-C, mmol/L				
Patients	2.7 (0.7)	2.7 (0.8)	2.5 (0.6)	ns
Controls	3.5 (0.8)	3.4 (0.9)	3.5 (1.0)	ns
LDL-C, mg/dL				
Patients	104.4 (27.1)	104.4 (30.9)	96.7 (23.2)	ns
Controls	135.3 (30.9)	131.5 (34.8)	135.3 (38.7)	ns
Microalbuminuria, n (%)				
Patients	3 (6.2)	27 (16.6)	30 (17.3)	ns
Controls	2 (2.7)	2 (2.4)	3 (11.1)	ns
Creatinine, µmol/L				
Patients	89.4 (12.8)	89.6 (18.1)	87.4 (15.8)	ns
Controls	73.3 (11.4)	80.6 (14.4)	85.9 (19.5)	I–II, I–III
Creatinine, mg/dL				
Patients	1.01 (0.14)	1.01 (0.20)	0.99 (0.18)	ns
Controls	0.83 (0.13)	0.91 (0.16)	0.97 (0.22)	I–II, I–III
GFR, mL/min/1.73 m^2^				
Patients	69.9 (8.9)	73.3 (17.1)	72.8 (16.4)	ns
Controls	80.0 (13.8)	76.9 (14.5)	72.9 (16.3)	ns
Systolic blood pressure, mmHg				
Patients	132 (19)	138 (15)	138 (17)	I–III
Controls	124 (18)	129 (18)	139 (13)	I–III
Diastolic blood pressure, mmHg				
Patients	76 (8)	79 (9)	77 (8)	ns
Controls	71 (9)	76 (11)	80 (8)	I–II, I–III
Echocardiography				
LA, mm/m^2^				
Patients	20.2 (2.3)	20.5 (2.3)	20.4 (2.3)	ns
Controls	20.9 (2.5)	20.5 (4.0)	19.6 (3.0)	ns
LVEDV, mL/m^2^				
Patients	50.2 (9.2)	48.9 (9.4)	46.6 (13.0)	ns
Controls	50.2 (9.2)	48.9 (9.4)	46.6 (9.9)	ns
LV mass, g/m^2^				
Patients	96.7 (20.6)	102.0 (22.5)	107.1 (26.4)	I–III
Controls	89.9 (16.9)	100.3 (22.9)	101.5 (32.4)	I–II
LVEF, %				
Patients	57 (8)	53 (8)	49 (9)	I–II, I–III, II–III
Controls	58 (6)	55 (7)	54 (8)	I–II, I–III
MAPSE, mm				
Patients	13 (2)	12 (2)	12 (2)	I–II, I–III
Controls	13 (2)	12 (2)	12 (2)	ns
GLS, %				
Patients	− 18.6 (2.3)	− 17.5 (2.3)	− 16.2 (3.0)	I–II, I–III, II–III
Controls	− 22.3 (3.0)	− 20.8 (3.1)	− 19.6 (4.0)	I–II, I–III
E/é				
Patients	11.9 (3.2)	14.0 (3.8)	14.7 (3.8)	I–II, I–III, II–III
Controls	10.3 (2.7)	11.3 (3.7)	12.5 (4.0)	I–III

Values are mean (standard deviation) values for continuous variables and numbers (percent) for proportions. LA, LVEDV and LV mass are indexed by body surface area. See abbreviations as in Tables 1 and 2

Overall, LVEDV and the E/é ratio increased from baseline to follow-up but on average, there was no change in LV mass or systolic function (Table 2). Patient weight was almost unchanged (increase 0.3 kg) and there were only minor changes in HbA1c, LDL, Triglycerides and creatinine. However, patients who gained weight (i.e. increased in weight ≥ 0.3 kg from baseline to follow-up) decreased their LVEF (median and interquartile range) by − 1.0 (9.0) % (n = 187) and patients who lost weight (i.e. dropped in weight by ≥ 0.3 kg) increased their LVEF by 1.0 (10.0) % (n = 179) (p < 0.05). There were no differences in changes in LV morphology, diastolic function or blood pressure between these two groups. In addition, changes in HbA1c, LDL, triglycerides and creatinine from baseline to follow-up could not explain changes in LV morphology or function.

Inter-observer variability in assessing left ventricle systolic function was determined towards a second, experienced echocardiographer, in 20 consecutive patients. The intraclass correlation (ICC), of LVEF was 0.86, MAPSE 0.95 and GLS 0.83. The coefficient of variation for LVEF was 5.0%, MAPSE 4.0% and GLS 6.9%. Interobserver bias and limits of agreement were 0.45 (− 4.79 to 5.69) for LVEF, − 0.01 (− 0.93 to 0.91) for MAPSE and 0.94 (− 1.46 to 3.34) for GLS.

## Discussion

The results of this long-term, observational study of middle-aged to elderly patients with T2DM and non-T2DM controls indicates that overweight and obesity are associated with impaired LV systolic function. Participants with overweight and obesity had increased LV mass, inferior LV systolic and diastolic function, as measured using LVEF, GLS and E/é, compared with the leaner participants. From both the univariable and multivariable regression analyses, we found that, irrespective of T2DM or other explanatory variables, overweight/obesity was the major risk factor for LV systolic dysfunction. Worsening LV systolic function was present at 4 years follow-up in the patients who had increased their BMI. These results are unique because they indicate the presence of an association between overweight/obesity and impaired LV systolic function, as measured by LVEF and GLS, in both a defined population with T2DM and a non T2DM control group.

### Obesity and LV diastolic function

Overweight and obesity were found to be associated with increased LV wall mass and the E/é ratio. This is consistent with the results of previous reports where obesity contributed to LV dilatation, hypertrophy, impaired relaxation, and diastolic dysfunction [3, 4, 8, 10]. Adiposity is associated with chronic volume overload resulting in increased peripheral vascular resistance. The Framingham Heart Study results indicated that obesity is associated with an increase in LV volume, wall thickness, and mass [23].

### Obesity and LV systolic function

Obesity is an established predictor of heart failure, but the extent to which it is associated with impaired LV systolic function is unclear. Previous studies have shown that it is associated with LV structural remodelling, LV diastolic dysfunction and lower longitudinal myocardial deformation, but not with reduced LVEF [6–8, 24]. Overweight has a greater effect on GLS in T2DM patients than in non-T2DM healthy subjects [7, 8]. Recently, abdominal adiposity, as measured by waist circumference, was found to be associated with impaired GLS but not decreased LVEF [10]. Our study results indicated a significant relationship between overweight/obesity and LV systolic function in terms of LVEF as well as GLS. Overweight/obesity and LVEF changes were even more closely associated than obesity and E/é changes. Several mechanisms, alone or in combination, may contribute to the impaired LV systolic function found in participants with overweight and obesity in our study. Patients with overweight and obesity had higher levels of HbA1c, compared with normal weight participants. Hyperglycaemia may contribute to LV mass independent of age, BMI, and blood pressure [25, 26]. High blood glucose levels induce excess intracellular calcium concentrations, which contribute to elevated blood pressure and an increase in LV mass [27]. Hyperglycaemia may increase intra- and extra-cellular glycation of proteins that increase oxidative stress, inflammation and myocardial damage, augmenting myocardial stiffness and reducing contractility [28]. However, the association between BMI and LV systolic function was also seen in the control group without T2DM implying that mechanisms different from what was previously enumerated contribute to worsening myocardial function. In our study population, patients with overweight and obesity had higher triglyceride levels than patients with normal weight which could have a bearing on previous findings that myocardial steatosis may impair LV systolic and diastolic function [29, 30]. Microalbuminuria is associated with elevated nocturnal blood pressures and LV systolic and diastolic dysfunction in T2DM patients [31]. Hypertension often coexists with diabetes, the synergistic effect on LV diastolic function is associated with LV hypertrophy and higher LV filling pressures. Advanced hypertension may cause LV systolic dysfunction and heart failure. The T2DM patients in our study had higher blood pressure than the controls, which could have contributed to the observed differences in LV function between the groups. The added effect of hypertension may accelerate the LV remodelling process in diabetic patients thus obscuring the effect of differences in BMI alone [32, 33]. The results suggest that hypertension has greater impact on LV mass and diastolic function than on LV systolic function.

The study design is crucial for what outcome can be expected. The association between obesity and LV systolic function demonstrated here may be different from what has been shown in other cohorts or in studies that excluded patients with known cardiovascular disease or reduced LVEF [6, 8, 10, 24].

What is the clinical relevance of impaired LV systolic function among persons with overweight and obesity in our study? Patients with T2DM and obesity had approximately 8% lower LVEF and controls with obesity about 4% lower LVEF than the normal weight participants. Corresponding figures for GLS were 2.4% vs 2.7%. Symptoms of heart failure are often nonspecific, but a reduction in LVEF is associated with increased comorbidity and mortality [15]. Therefore, the impairment in LV systolic function, associated with overweight and obesity in our study is important. It may indicate an increased risk of cardiovascular events and thus a need for intensified metabolic control, adjustment of blood pressure as well as weight reduction to prevent overt heart failure [34].

How valid are the LVEF results in our study? The speckle-tracking based Auto-EF method has been demonstrated to be reliable compared with MRI. It was also faster and had lower inter- and intra-observer variability than manual assessment by the use of the modified Simpson’s rule [17]. The inter-observer variability was low in our study which supports the view that the measurements are valid.

We propose that, compared with normal weight persons, individuals with overweight or obesity are more prone to develop LV systolic dysfunction as measured by use of LVEF and GLS.

### Long-term results

LV end-diastolic volumes and E/é values increased 4 years after the initial assessment, indicating the presence of elevated filling pressures to the left ventricle and progressive LV diastolic dysfunction. LVEF decreased in participants who increased in weight from baseline to follow-up which further supports our finding of an association between obesity and LV systolic dysfunction. This study was designed as an observational study. We did not intend to improve the already appropriate medical care of the participants and could not identify a subgroup of patients that improved cardiac function. However, better glycaemic control may contribute to regression of LV hypertrophy in patents with T2DM [35].

### Study limitations

Our study had some limitations. There were fewer women than men in the patient population. Men with obesity are more likely to be affected by concentric cardiac hypertrophy than eccentric hypertrophy while women with obesity experience both types of hypertrophy [36]. The difference in LVEF between groups and the change over time is small and the clinical effect was not studied. Some laboratory results were missing in individual participants in the follow-up study. Left atrial volume and left ventricle mass were assessed by use of the M-mode technique and not from measurements in 2D or 3D that would have been preferable. Tissue velocity was recorded with color tissue Doppler instead of using pulsed tissue Doppler.

## Conclusions

Overweight and obesity were associated with LV structural remodeling and dysfunction in middle-aged to elderly patients with T2DM as well as in their non-diabetic controls. Compared to participants with normal weight, individuals with overweight and obesity had impaired LV systolic function as measured by both LVEF and GLS. Progression in LV remodelling was seen at the 4-year follow-up in T2DM patients who had further gained weight and increased their BMI. Our study reveals that overweight and obesity are major risk factors for impaired LV systolic function.

## Abbreviations

ACEI: angiotensin converting enzyme inhibitor
ARB: angiotensin II receptor blocker
BMI: body mass index
BSA: body surface area
CARDIPP: Cardiovascular Risk factors in Patients with Diabetes—a Prospective study in Primary care
CAREFUL: Cardiovascular Reference Population study
E: transmitral E-wave velocity
eʹ: early diastolic mitral annulus velocity
GLS: peak global longitudinal strain
HbA1c: glycosylated haemoglobin
HDL: high density lipoprotein
ICC: intraclass correlation
LA: left atrium
LDL: low-density lipoprotein
LV: left ventricle
LVEDD: left ventricular end-diastolic diameter
LVEDV: left ventricular end-diastolic volume
LVEF: left ventricular ejection fraction
MAPSE: mitral annular plane systolic excursion
SD: standard deviation
T2DM: type 2 diabetes mellitus
